# A comparative analysis of computer based hospice palliative care datasets in Canada

**DOI:** 10.1186/1472-684X-7-6

**Published:** 2008-05-12

**Authors:** Craig E Kuziemsky, Francis Lau

**Affiliations:** 1 Telfer School of Management, University of Ottawa, 55 Laurier Avenue East, Ottawa, Ontario K1N 6N5, Canada; 2 School of Health Information Science, University of Victoria, PO Box 3050 STN CSC Victoria, BC V8W 3P5, Canada

## Abstract

**Background:**

To analyze how seven Canadian hospice palliative care (HPC) centres and one national surveillance dataset compare with respect to the collection of forty data elements. Research and service delivery implications of the findings are discussed.

**Methods:**

The data sources consisted of data element names and their definitions collected in a computer based format from seven HPC centres and one surveillance dataset. The data elements were structured into five themes: demographic, patient death, support, contact or informal caregiver; program/consultations/service request, and clinical. Each theme contains a number of data elements with a total of 40 elements included in the analysis. Comparative analysis was done on the data elements to compare their names and definitions.

**Results:**

Much variation exists in data collection around HPC delivery. Such variation prevents any timely and meaningful comparison of service and care delivery across HPC centres. Patient death data, service/program data and clinical data is particularly varied.

**Conclusion:**

Developing a common minimum data set is a logical starting point to help overcome data variations between care centres. Greater coordination is needed between care centres and the development of national standards and policies. Moving towards electronic data collection would help facilitate common policy and practice norms.

## Background

Although modern hospice palliative care (HPC) is still an emerging field of medicine, our aging population and increased onset and survival time of patients with chronic illness will increase the need for access to HPC services. In Canada there have been national initiatives over the past five years including a 2000 report from the Senate Subcommittee for End-of life Care [1] and its 2005 follow up [2] that have acknowledged both the need for increased HPC delivery as well as the need for new approaches to delivering HPC.

As a response to such initiatives Canada has seen an increase in HPC programs, and the ability to deliver HPC. HPC is delivered through a number of venues including acute care hospitals, long-term care centres and specialist HPC services such as hospices, and homecare services. Along with variations in where and how HPC is delivered are variations in what data is collected to support care delivery. In the absence of common guidelines or data collection strategies HPC centres have established local datasets to meet their individual needs, which has resulted in wide variation in what data is collected. HPC data can be structured at three levels, clinical (data that represents individual patient management and decision making such as symptoms and medications), program/service (data that represents a program or unit and informs decision making such as staffing and resource needs) and surveillance (data that represents high levels comparison such as the range of locations or length of time that patients access services and is used for provincial or national level decision making). Although there has been research to study data collection across different centres much of it has focused on surveillance level datasets. An example is Gaudette et al. [3] where they describe a pilot study for establishing a national hospice palliative surveillance system in Canada. Surveillance studies are useful; however they only provide one view of HPC delivery. Many data elements that are part of HPC such as detailed service delivery, program and clinical data elements are excluded from surveillance studies. While studies exist that analyze all three levels of HPC data from individual hospice palliative care centres there is little research that compares the levels of data across different centres.

In Canada there is ongoing work at developing national standards for HPC delivery. Examples of such work include the 2006 Canadian Council of Health Service Accreditation (CCHSA) Hospice palliative Care and End of Life Care Standards [4] and the 2006 Canadian Hospice Palliative Care Association (CHPCA) Gold Standards for Hospice palliative Home Care [5]. In order to implement and compare congruence with such standards we will require data from all three levels of hospice palliative care delivery. However there are logistical issues with both the collection and analysis of data from HPC centers. One comment from the surveillance pilot project by Gaudette et al. [3] was that differences in data formats from the various centres made the analysis difficult. There are two needs that would enhance our ability to perform meaningful data analysis: a common framework for data collection and an efficient means of data collection and analysis. The first need, a common framework for data collection, could be achieved by developing a minimum data set (MDS). Examples of hospice palliative MDS include the National Data Set in the United States [6], the National Council for Hospice and Specialist Palliative Care Services MDS in the United Kingdom [7], and the National Minimum Data Set for hospice palliative care in Australia [8]. The above MDS have allowed meaningful understanding around HPC access, delivery and outcomes [6,7] and [9].

The second need, efficient data collection and analysis, could be served by computer based data collection and the development of HPC databases to house the data. Although much of hospice palliative data is currently collected in paper based format there is much value to be obtained by moving towards computer based data collection. Computer based data would enable easier collation and comparison of data between centres and also enable us to move towards a national hospice palliative database to allow ongoing collection and comparison of data across centers and with national standards such as the aforementioned CCHSA and CHPCA standards.

Canada has yet to implement a national HPC MDS but given that many HPC programs or centres are assessing their data needs and beginning to develop data sets for their own use it would be ideal to have a MDS to serve as a common framework for centers to develop their datasets. It is crucial that Canada have a well thought out strategy for the collection and analysis of HPC data as the data we collect will provide the answers to questions about how HPC services are being delivered and accessed. The answers to those questions will shape our ability to advocate for funding and policy development in the forthcoming years. Thus we need to ensure the answers we provide are accurate and meaningful.

This paper provides a comparative analysis of how seven Canadian HPC centres and one national surveillance dataset compare with respect to the collection of forty data elements. For the seven centers we have chosen to only compare the names and definitions of data that are currently collected in a computer based format. The forty elements span all three levels of HPC delivery and include surveillance, service/program delivery and clinical level data elements. The paper is an important first step towards understanding what data is used in Canada at all levels of hospice palliative care delivery.

The three main purposes of this paper are:

1. Compare the data element names and, where available, their definitions of seven Canadian HPC centres and one national surveillance dataset.

2. Provide some preliminary analysis of the data element names and definitions to identify similarities and differences around what type of data is collected.

3. Suggest means of improving how data is collected to enhance HPC delivery.

The scope of this analysis covered only the data element names and their definitions (if provided by the centres) collected in computer based format, and did not include any comparison of actual patient data or codes that are collected.

## Methods

In 2004, the authors made requests to seven HPC centres across Canada to obtain information on their existing palliative care databases in order to conduct a comparative analysis. The information consisted of the data element names and their definitions that are collected in a computer based format from the seven HPC centres. This process took several iterations over two years since some data elements required clarification/confirmation and others were updated since the initial request was made. The seven centres and date the data element names and definitions were obtained are Calgary Health Region Hospice Palliative Care Program, Calgary AB (2005); Capital Health Hospice Care Program, Halifax NS (2004); Capital Health Regional Palliative Care Program, Edmonton AB (2005); Queen's Palliative Medicine Program, Kingston ON (2002); Temmy Latner Centre for Hospice Care, Toronto ON (2002); Victoria Hospice Society, Victoria BC (2003); and Winnipeg Regional Health Authority Palliative Care Sub Program, Winnipeg MB (2004). The seven centers provide similar types of care in that all provide both inpatient (i.e. hospice unit or acute care consultations) and community based care. The data element names and definitions from the Health Canada Surveillance Data Set (SDS) (2002), which represents a consensus of centres participating in a national surveillance pilot study, were also included to illustrate the surveillance data and also to illustrate the range of data elements that are not represented in surveillance studies.

Because our analysis is only on the type of data defined and collected in a computer based format it is likely that some of the data elements that are not indicated as being collected by a centre are captured in paper charts or through information systems at the organizational level but not integrated with the HPC databases.

## Results

The results consist of a comparison of the data elements defined and collected from the seven centres and the Health Canada SDS. In order to enhance the comparability of these data elements five themes were devised: demographic, patient death, support, contact or informal caregiver; program/consultations/service request, and clinical. These five themes represented logical groupings of the data elements but the themes are consistent with classes of data elements collected in common hospice palliative care data sets such as the National Data Set in the United States and the National Council for Hospice and Specialist Palliative Care Services MDS in the United Kingdom. Each theme contains a number of data elements with a total of 40 data elements included in the analysis.

The results are presented as a separate section for each of the five themes. Each section consists of a table and a discussion of the results. The tables list the HPC centres along the horizontal axis and the data elements along the vertical axis. A check mark indicates whether the data element is collected at each of the seven centres or in the Health Canada SDS.

### Demographic data

Demographic data (Table 1) comprises much of what is collected in surveillance studies and therefore is well captured by the Health Canada SDS. Religion, ethnicity, lives alone and communication data (i.e. patient address or telephone) are the only demographic element not captured by the Health Canada SDS and in fact religion is only collected by two centres (Toronto and Halifax). The main differences in demographic elements are with elements 4–6 and 9. Again because demographic data is commonly captured in hospital registration systems some of the missing elements are likely captured elsewhere. It should be noted that variations of Lives Alone/Lives With data are also captured as spousal information in the Support, Contact or Informal Caregiver information. All datasets (including the Health Canada SDS) capture some form of patient ID although capturing more than one was common. For instance most centres captured both a provincial health care number and a hospice palliative program ID.

**Table 1:** Demographic Data

	**Calgary**	**Edmonton**	**Halifax**	**Kingston**	**Toronto**	**Victoria Hospice**	**Winnipeg**	**Health Canada SDS **
1. Patient ID	✓	✓	✓	✓	✓	✓	✓	✓
2. Address	✓ (city only)	✓	✓	✓	✓	✓	✓	✓ ^1^
3. Date of birth	✓	✓	✓	✓		✓	✓	✓
4. Religion			✓		✓			
5. Ethnicity					✓			
6. Spoken Language					✓			✓
7. Marital Status		✓		✓	✓			✓
8. Communication means (i.e. phone/fax)		✓	✓	✓	✓	✓	✓	
9. Lives Alone/Lives With	✓				✓			

^1^Health Canada collects province and postal code (either 3 or 6 digits depending on location).

### Patient death data

The capture of patient death information (Table 2) is varied across the centres. Date and location of death are the only data collected consistently with much variation in the other patient death elements. Given the expansion of home based HPC and more patients wanting to die at home we need to capture more informative data around patient deaths. One option for obtaining these data fields in a consistent manner is to have an ongoing link between hospice palliative care datasets and Vital Statistics death certificate data. One data element that is necessary is detail on where a patient would prefer to die and whether or not their preference was realized. Currently only Winnipeg collects data around whether a home death is possible or not and thus further studies and data capture around home deaths and the context of which they occur is needed. Halifax and Toronto capture currently capture data on death wishes for the patient and family. Only Toronto captures data on whether the patient/family death wishes were kept. Halifax collects the place of death desired by the patient and the patient's current location; therefore it may be possible to infer whether the patient died in their preferred location.

**Table 2:** Patient Death Data

	**Calgary**	**Edmonton**	**Halifax**	**Kingston**	**Toronto**	**Victoria Hospice**	**Winnipeg**	**Health Canada SDS**
10. Date of Death	✓	✓	✓	✓	✓	✓	✓	✓
11. Time of death						✓	✓	
12. Location of death			✓	✓	✓	✓	✓	✓
13. Patient/family death wishes captured?			✓		✓			
14. Patient/family Wishes Kept					✓			
15. Home Death not possible?							✓	
16. Bereavement Details			✓			✓	✓	

### Support, contact or informal caregiver data

There is good consistency in the capture of support, contact or informal caregiver data (Table 3), with the exception of Calgary and the Health Canada SDS. Calgary does capture whether the patient lives alone (shown in the demographic Table 1), which also provides an indication of potential support resources that may be available. Calgary in fact captures details such as whether the patient lives alone, with others, with a spouse, with a spouse as well as others or with other family members.

**Table 3:** Support, Contact or Informal Caregiver Data

**Data Element**	**Calgary**	**Edmonton**	**Halifax**	**Kingston**	**Toronto**	**Victoria Hospice**	**Winnipeg**	**Health Canada SDS**
17. Name		✓	✓	✓	✓	✓	✓	
18. Relationship		✓	✓		✓	✓	✓	
19. Contact Info (i.e. telephone)		✓	✓	✓	✓	✓	✓	

Currently Toronto is the only centre to capture extensive data around informal caregivers. Toronto captures data on the health status and capabilities for care delivery from informal caregivers. Further information about support, contacts or informal caregivers will be needed as more home based hospice palliative care is delivered. In particular it will become important to make a distinction between residing with someone and residing with someone who is able to act as an informal caregiver. Numerous studies have illustrated the impact of providing care on informal caregivers and as informal caregivers play increasingly important roles in care delivery we will need to ensure such data is captured.

### Program/consultations/service request data

Program/Consultations/Service Request data is presented as three sub-themes: program data (Table 4), consultations (Table 5) and service requests and events (Table 6).

**Table 4:** Program Data

	**Calgary**	**Edmonton**	**Halifax**	**Kingston**	**Toronto**	**Victoria Hospice**	**Winnipeg**	**Health Canada SDS**
20. Date of program registration	✓	✓	✓	✓	✓	✓	✓	✓
21. Referred By			✓	✓	✓	✓	✓	
22. Program Discharge Date	✓	✓	✓		✓	✓	✓	✓
23. Location to which Discharged	✓	✓	✓		✓			✓

**Table 5:** Consultation Data

	**Calgary**	**Edmonton**	**Halifax**	**Kingston**	**Toronto**	**Victoria Hospice**	**Winnipeg**	**Health Canada SDS**
24. Date of consult registration			✓	✓	✓	✓	✓	
25. Type of consult (i.e. RN, MD, counselling)			✓	✓	✓	✓		
26. Location of Consult			✓		✓	✓		
27. Referred By				✓	✓	✓		

**Table 6:** Service Requests & Events Data

	**Calgary**	**Edmonton**	**Halifax**	**Kingston**	**Toronto**	**Victoria Hospice**	**Winnipeg**	**Health Canada SDS**
28. Date of request	✓			✓	✓	✓		
29. Priority/Urgency			✓		✓	✓		
30. Requested By	✓				✓	✓		
31. Service Requested	✓		✓	✓	✓	✓		
32. Date of Response	✓		✓	✓	✓	✓		
33. Time of Response				✓	✓	✓		

The program/consult/service request distinctions were made to help make sense of how patients are registered into HPC programs and how services are provided. Program data refers to the overall HPC program. Once a patient is registered in the program they would receive services until such time they are discharged. Services could be a homecare visit or admission to a HPC unit. The consult category is derived from our experiences in Victoria, BC with Victoria Hospice. Victoria Hospice captures consults as services provided to patients who are not registered patients in the VHS palliative program. For example a Victoria Hospice physician may see a patient in an acute care hospital and that would be recorded as a consult visit.

This section was the most difficult to do comparative analysis on as there is the largest degree of variance between data. We recognize that each centre has its own protocol for registering patients and tracking consults/services and thus some centres may not fit the program, consult and service model we have devised. Therefore if a centre is shown as not collecting a particular data element it may be because the element is rolled up within another element such as consult data being recorded within service data. Also, in some cases a centre may collect an element related to 'urgency/priority' but it is unclear whether it referred to urgency of service, program or consult. Since our analysis only represents urgency/priority in the service table (Table 6) we showed a centre as collecting urgency/priority if their data contained some type of an urgency data element. That ambiguity emphasizes the need for further research around how program/service/consult data are defined and used in practice. The analysis of program/consults/services is where having the data definitions was particularly useful as it allowed us to understand differences in how terms such as program or service are defined. For example Halifax refers to a service as a hospital service a patient moves to or from such as ICU, Orthopedics or Surgery whereas Victoria Hospice refers to a service in the context of HPC service such as a HPC response team visit or hospice physician consult. So although both Halifax and Victoria Hospice have the term service in their database they have quite different meanings.

Because HPC patients often move through many different care settings most centres attempt to track patients as they move across different care settings, such as if a patient moves from being admitted in a hospice palliative care unit to home care. The Health Canada SDS made a point of attempting to capture multiple admissions by the same patient to different HPC programs/settings by tracking patient movements across care settings. A different approach is used by Halifax in that they have a database table called transitions to store the movement of a patient from one setting to another. A transition record tracks the location a patient goes to and from, the service they will get at the new location and the service they are being move from.

### Clinical data

The collection of clinical data (Table 7) shows much variation with the patient's clinically coded primary disease/diagnosis being the only data element common across all centres. All centres expect for Halifax and Winnipeg capture comorbidites. Halifax, Toronto, Victoria, Health Canada SDS and Calgary all capture diagnosis using the International Classification of Disease (ICD) coding system. The data element for whether the patient is aware of the HPC prognosis is the least captured element with Toronto being the only centre collecting it. Other examples of clinical data elements collected include the Edmonton Symptom Assessment System (ESAS), and Mini-mental State Examination (MMSE), the Palliative Performance Scale (PPS). The type, timing and frequency of assessment being done varied between HPC centres. For instance, The Capital Health Regional Palliative Care Program in Edmonton collects ESAS, MMSE and PPS on a regular basis; whereas the Victoria Hospice routinely collects PPS only.

**Table 7:** Clinical Data

	**Calgary**	**Edmonton**	**Halifax**	**Kingston**	**Toronto**	**Victoria Hospice**	**Winnipeg**	**Health Canada SDS**
34. Disease	✓	✓	✓	✓	✓	✓	✓	✓
35. Diagnosis Date			✓	✓	✓		✓	✓
36. Co-morbidity	✓	✓		✓	✓	✓		✓
37. Allergies				✓	✓	✓		
38. Drugs & medication (i.e. dose, route)		✓		✓	✓	✓		
39. Patient aware of hospice palliative care prognosis					✓			
40. Symptom Assessments (all symptoms including pain)	✓	✓			✓	✓		

## Discussion

This paper has presented a comparative analysis of the data element names and definitions that are collected in computer based format by seven Canadian HPC centres and one non-computer based surveillance dataset. The results provide important groundwork for understanding the types of data that is collected and where data collection differences occur between hospice palliative centres in Canada. The results showed that we need improved data collection in a number of areas including referral and access to HPC and informal caregivers or supports and their ability to provide care and the context and appropriateness of home deaths. When patients die at home it is often unknown whether the patients are dying at home through choice, a lack of services preventing them from dying elsewhere, or inefficient communication or referral about available services that would allow them to die elsewhere. Although national initiatives in Canada are advocating increased HPC delivery we first need to be able to answer basic questions such as who is and is not accessing HPC and perhaps most importantly why are patients not accessing HPC? However before we can answer those and other questions we need to collect common data to enable comparisons and analysis to inform policy development. One significant finding from the results is that some HPC centres collect what appears to be a similar data element but in reality the elements are quite different. For example Halifax and Victoria both collect data on services but the definitions of services are quite different at the two centres.

HPC in Canada is somewhat at a crossroads in terms of the development of standards and practice norms and the development of HPC and end-of-life programs where care is provided. There has been some significant research done establishing standards and practice norms for hospice palliative care, such as the aforementioned CCHSA standards and the CHPCA norms of practice [10]. The Resident Assessment Instrument for Palliative Care (RAI-PC) [11] is also starting pilot tests in Canada and that tool will also have impacts on what data is collected by HPC programs. One key issue to date is that the development of HPC programs and the establishment of models and norms of practice have largely occurred in a fragmented rather than coordinated manner. That lack of coordination limits our ability to see the extent by which such standards are implemented, which also prevents us from evaluating their effectiveness in enhancing HPC delivery. Existing standards and norms of practice for HPC and assessment tools such as RAI-PC need to be considered and ideally consulted as palliative datasets are developed. Further, healthcare is increasingly becoming more computer based through the development of electronic health record and health information systems. Data is the lifeblood of both of those types of systems and the data needs of HPC must be defined and brought forward otherwise HPC is in danger of missing its opportunity to influence the design of information and communication technologies that will shape how data is collected and disseminated in future healthcare delivery.

Research steps arising from this paper include the need to work towards development of a common MDS for HPC in Canada. A common MDS would enable research to be done about the provision of HPC in Canada such as comparing length of stay, access to and use of hospice palliative services and management of symptoms. We cannot strictly rely on surveillance level MDS however as this study showed that many of the service delivery and clinical data elements were missing from the Health Canada SDS. Part of this MDS should also include HPC outcome data. Examples are the extent of symptom control achieved such as pain and dyspnea as a result of the clinical assessments and interventions provided. Others may include the fulfillment of patients' wishes such as preferred care settings and support options [9].

However in developing an MDS Fainsinger and Fassbender [12] correctly point out that a theoretical framework needs to be considered as in the past data sets have been developed more on availability of data rather than need or research usefulness. In particular it is imperative that such a theoretical framework be based upon standards and practice norms from key organizations such CHPCA, CCHSA and CIHI (Canadian Institute for Health Information). Given that Canada is still in early stages at developing a common hospice palliative data set it makes sense to draw upon existing work in other countries including the aforementioned National Data Set in the United States, National Council for Hospice and Specialist Palliative Care Services MDS in the United Kingdom and the National Minimum Data Set for HPC in Australia. Ideally as our MDS work in Canada progresses we need to compare our findings to existing HPC research so we can learn from each other and foster a global environment of enhanced HPC delivery.

A further research issue is that data analysis studies generally have a long time span between collection of data, analysis of results and dissemination of findings. What is needed is a means of getting results into practice in an expedited manner that also allows analysis results to be continuously updated to reflect the current state of HPC delivery. In that regard researchers at the University of Victoria have begun work on an electronic database translation engine for automated comparison of one HPC database to another [13]. Such a tool would allow HPC centres to submit anonymized data electronically to a data warehouse so the data could be analyzed against data from other centres. Centres would then be able to request reports showing how their data compares to other centres. Such analysis would allow comparisons of hospice palliative care programs to enable programs to develop common policy and practice norms.

The limitations of our study are that we only focused on data collected in computer based format. That was done partially because we had access to the database schemas of the seven HPC centres and also because we believe HPC data collection needs to move towards electronic collection. While we were able to compare the data element names and definitions provided by these HPC centres, we did not have access to the actual patient data collected. Therefore, we were not able determine the extent to which the specified data elements were actually being collected or the completeness or reliability of data collection. Further research is needed that compares the actual data that is collected across different centres and looks at issues of data quality. Research is also needed to map the data elements to formal medical terminologies such as SNOMED-CT as that would enable the formal terminology to act as a standard term to facilitate comparative analysis. Standard terms would also promote better data quality.

Also, we have not included data that is collected at regional or health authority levels as we believe it made sense to start with data at the HPC program level as that is where data collection originates. Further studies will include looking at actual patient, regional and health authority data as well as incorporating costing data into our analysis.

## Conclusion

This paper has shown that there are similarities but also key differences in how HPC data is collected in Canada. The paper also identified further research that is needed such as linkages between conceptual models and practice delivery and a better understanding of care delivery in areas such as home deaths and informal caregivers. However a logical starting point is to continue our drive towards a common hospice palliative care MDS. That will enable us to compare data and outcomes from the data, which will help our drive towards establishing practice norms and ensure that HPC informatics continues to evolve.

## Competing interests

The authors declare that they have no competing interests.

## Authors' contributions

CEK obtained the data and did the initial analysis and compilation of results. CEK & FL did a joint review of the results that CEK compiled into an initial draft of the manuscript. Both authors read and approved the final manuscript. Revisions based on reviewer feedback were done by both CEK & FL. Both authors have read and approved the final version of the manuscript.

## Pre-publication history

The pre-publication history for this paper can be accessed here:


